# Tumor Microenvironment Proteomics: Lessons From Multiple Myeloma

**DOI:** 10.3389/fonc.2021.563384

**Published:** 2021-03-23

**Authors:** Rodrigo Carlini Fernando, Fabrício de Carvalho, Adriana Franco Paes Leme, Gisele Wally Braga Colleoni

**Affiliations:** ^1^ Department of Experimental and Clinical Oncology, Discipline of Hematology and Hemotherapy, Federal University of São Paulo, UNIFESP, São Paulo, Brazil; ^2^ Laboratory of Mass Spectrometry, Laboratory of National Biosciences, LNBio, National Council for Research in Energy and Materials (CNPEM), Campinas, Brazil

**Keywords:** multiple myeloma, tumor microenvironment, proteome, proteomics, mass spectrometry

## Abstract

Although the “seed and soil” hypothesis was proposed by Stephen Paget at the end of the 19th century, where he postulated that tumor cells (seeds) need a propitious medium (soil) to be able to establish metastases, only recently the tumor microenvironment started to be more studied in the field of Oncology. Multiple myeloma (MM), a malignancy of plasma cells, can be considered one of the types of cancers where there is more evidence in the literature of the central role that the bone marrow (BM) microenvironment plays, contributing to proliferation, survival, migration, and drug resistance of tumor cells. Despite all advances in the therapeutic arsenal for MM treatment in the last years, the disease remains incurable. Thus, studies aiming a better understanding of the pathophysiology of the disease, as well as searching for new therapeutic targets are necessary and welcome. Therefore, the present study aimed to evaluate the protein expression profiling of mononuclear cells derived from BM of MM patients in comparison with these same cell types derived from healthy individuals, in order to fill this gap in MM treatment. Proteomic analysis was performed using the mass spectrometry technique and further analyses were done using bioinformatics tools, to identify dysregulated biological pathways and/or processes in the BM microenvironment of patients with MM as a result of the disease. Among the pathways identified in this study, we can highlight an upregulation of proteins related to protein biosynthesis, especially chaperone proteins, in patients with MM. Additionally, we also found an upregulation of several proteins involved in energy metabolism, which is one of the cancer hallmarks. Finally, with regard to the downregulated proteins, we can highlight mainly those involved in different pathways of the immune response, corroborating the data that has demonstrated that the immune system of MM is impaired and, therefore, the immunotherapies that have been studied recently for the treatment of the disease are extremely necessary in the search for a control and a cure for these patients who live with the disease.

## Introduction

Multiple myeloma (MM) is a hematologic malignancy of post-germinal center plasma cells, characterized by the infiltration of these tumor cells in the bone marrow (BM), presence of clonal immunoglobulin in the blood and/or urine of most patients, and damage to target tissues and organs, including bone lesions, hypercalcemia, kidney failure and anemia, and an increased risk of infections (1, 2). MM accounts for approximately 1% of all cancer cases and 10% of hematologic malignancies, being the second most common hematological malignancy after lymphomas (3). Over 30,000 new patients are diagnosed in the United States each year (4). The average age at diagnosis is 70 years and only 2% of patients are less than 40 years of age at diagnosis (4), which makes this disease attractive to study the interactions among tumor immunology, immunosenescence and aging, since the majority of the new cancer cases are diagnosed in people aged above 60 (4).

MM etiology is unknown, but aging and life exposure to cancer risk factors, including environment exposure to carcinogens, such as radiation, might contribute to MM development (5). Men are slightly more affected than women (4), Afro-descendants are twice as affected compared to Caucasians (4), and almost all cases of MM evolve from a premalignant condition called monoclonal gammopathy of undetermined significance (6, 7).

The treatment of MM has evolved markedly over the past few years, especially due to the introduction of high-dose of melphalan followed by autologous stem cell transplantation for eligible patients, and the introduction of immunomodulatory drugs such as thalidomide and lenalidomide, and proteasome inhibitory molecules such as bortezomib (8). More recently, several new drugs have been approved, including new immunomodulators (pomalidomide) and proteasome inhibitors (carfilzomib, ixazomib), improving the risk-benefit profile of these classes of therapies (9). Besides, monoclonal antibodies (anti-CD38 and anti-SLAMF1) was also introduced in the therapeutic MM armament, and an inhibitor of the histone deacetylase enzyme (panobinostat) (9).

These new molecules had a major impact on the survival of patients and currently younger individuals diagnosed with MM can have an average survival of ten years (10). New approaches involving immunotherapies such as CART (chimeric antigen receptor T)-cells or bi-specific antibodies (BiTEs) are underdevelopment, although they will not be available for the majority of patients worldwide (11). However, although all this progress has occurred with the expansion of the therapeutic arsenal, most patients will relapse one or more times over the course of the disease and, eventually, will become refractory to treatments available. Therefore, MM remains an incurable disease and further studies are needed to better elucidate the pathophysiology of the disease, as well to discover new potential therapeutic targets and surrogate biomarkers.

With regard to the pathophysiology of MM, BM tumor microenvironment plays a central role during disease development, and in its maintenance and progression (12). A growing body of evidence has shown that this tumor microenvironment, consisting of cellular and non-cellular elements, differs in its composition when comparing MM patients and healthy individuals (13). Regarding the cellular elements, we can highlight the mesenchymal stem cells (MSC), which have the capacity of self-renewal and differentiation in several cell types such as fibroblasts, osteoblasts, adipocytes, chondrocytes, among others (14). Through direct and indirect communication with tumor plasma cells, MSC promote their proliferation, survival, migration and drug resistance (15). Additionally, several studies, including one conducted by our group, have shown that MM MSCs differ in several aspects, including, gene expression and functional changes, in comparison to their normal counterparts derived from BM of healthy individuals (16). Other cell types are also very important to MM progression, including myeloid derived immunosuppressive cells (MDSCs), T cells, NK cells, dendritic cells, macrophages, osteoclasts and osteoblasts (13). These cells contribute to the maintenance of a tolerogenic microenvironment in BM, favoring the evasion of the immune system by tumor plasma cells. Besides, the imbalance of proliferation, differentiation and function of osteoblasts and osteoclasts are one of the most important factors for the formation of bone lesions in these patients. With regard to the non-cellular elements of the tumor microenvironment, we can mention cytokines, chemokines, growth factors and exosomes, which favor the progression of the disease, in addition to an altered composition of the extracellular matrix, which also contributes to the pathophysiology of MM (13).

Among the current therapies approved for MM, some of them also have effect on the tumor microenvironment mentioned above (17). Thus, for a better understanding of the biology of the disease and the search for new therapies, studies on the BM microenvironment of these patients are extremely relevant. In this context, proteomic studies are a very interesting approach to characterize the protein expression profiling of these cells from MM compared to BM cells from healthy individuals. In addition, evidences from the literature show that proteomic analysis gives a high confident picture of proteins differentially expressed, when compared with gene expression analysis (18).

Therefore, the present study aimed to evaluate the protein expression profiling of cells derived from BM of MM patients in comparison with these same cell types derived from healthy individuals, in order to search for potential new therapeutic targets and surrogate biomarkers, as well as to better understand the pathophysiology of the disease.

## Casuistic and Methods

### Ethical Aspects

This study was approved by the Institutional Review Board from Federal University of São Paulo (CAAE: 07297019.9.0000.5505). BM samples from MM patients and healthy donors were obtained after written informed consent forms by participants or legal representative, according to Helsinki Declaration and local regulations.

### Subjects

Eight newly diagnosed patients with MM (and without any previous treatment for the disease) were successfully enrolled in this study and allocated in the case group. Six young healthy individuals (BM donors for allogeneic stem cell transplantation) not matched by age or gender were also enrolled in this study and allocated in the control group.

### Bone Marrow Samples and Sorting of Mesenchymal Stem Cells and Mononuclear Cells

BM samples from newly diagnosed MM patients (n=8) and healthy donors (n=6) were harvested from iliac crest, through core biopsy marrow aspiration. Then, BM mononuclear cells were isolated by density gradient, using Ficoll-Paque PLUS (GE Healthcare, Little Chalfont, Bucks, GBR) and following the manufacturer’s instructions. Firstly, plasma cells were sorted by Magnetic-Activated Cell Sorting (MACS) methodology, using CD138^+^ as a positive marker (Miltenyi Biotec, Bergisch Gladbach, DEU). These cells were analyzed in another study from our group (19). Then, MSC were isolated, using CD105^+^ as a positive marker. Due to the low number of these cells, both in patients’ and controls’ BM, they were expanded *in vitro* according to the protocol described in Fernando et al. (16), n=4 for each group. Finally, the flow through, i.e., the BM mononuclear cells depleted of plasma cells (CD138^+^) and MSC (CD105^+^) were frozen in liquid nitrogen for the following experiments. In this study, we named these cells as mononuclear cells (MC)^CD138-CD105-^.

### MSC Characterization

After MSC *in vitro* expansion, we characterized the MSC according to the protocol described in Fernando et al. (16). In summary, positive markers CD105, CD90 and CD73, and negative markers CD45, CD34, CD14, and HLA-DR were tested by flow cytometry. In addition, the osteoblastic differentiation capacity of these cells was also evaluated, by measuring the osteocalcin protein produced by these cells and secreted in the culture medium supernatant on days 7, 14 and 21, during the stimulus that favors the differentiation of them into osteoblasts. The Human Osteocalcin Quantikine ELISA kit (R&D Systems, Minneapolis, MN, USA) was used to perform this analysis, according to the manufacturer’s instructions. All MSC from both groups used for characterization and submitted to mass spectrometry were between passages 2 to 5 of cell culture.

### Protein Extraction and Quantification

We pooled the samples from each group in order to obtain the required amount of protein to perform the mass spectrometry experiment. Therefore, we obtained **1)** a pool from MC^CD138-CD105-^ (n=8) and **2)** a pool from MSC (n=4), all derived from MM patients, i.e., four MM patients had both the MC^CD138-CD105-^ and the MSC analyzed by mass spectrometry; and **3)** a pool from MC^CD138-CD105-^ (n=6) and **4)** a pool from MSC (n=4), all from healthy donors (HD), i.e., four HD had both the MC^CD138-CD105-^ and the MSC analyzed by mass spectrometry. Protein extraction and quantification were performed using the Pierce Surface Protein Isolation kit and the Pierce 660 nm Protein Assay, respectively (Thermo Scientific, Rockford, IL, USA), following the manufacturer’s instructions.

### Mass Spectrometry Analyses

The proteins (30μg) were reduced (5 mM dithiothreitol, 25 min at 56°C), alkylated (14 mM iodoacetamide, 30 min at room temperature in the dark), and digested with trypsin (Promega). The resulting peptides were submitted to desalting using C-18 solid phase extraction cartridges (Sep-Pak^®^ Vac tC18 cartridge 3cc/100mg, Waters) and resuspended in dissolution buffer of iTRAQ 4-plex kit. The samples were labeled following the manufacturer’s instructions: MM-MC^CD138-CD105-^ was labeled with the 117 isobaric tag and the HD-MC^CD138-CD105-^ with the 116. As each sample was evaluated in a technical replicate, they were also marked with the other isobaric tag, i.e., the cells from MM patients with the tag 116 and the controls with tag 117. The same rationale was used for MM-MSC and HD-MSC labeling, but, in this case, isobaric tags 114 and 115 were used.

The resulting peptide mixture (5 μg) was analyzed on an ETD enabled LTQ Velos Orbitrap mass spectrometer (Thermo Fisher Scientific) coupled with LC-MS/MS by an EASY-nLC system (Proxeon Biosystem) through a Proxeon nanoelectrospray ion source. Peptides were separated by a 2-90% acetonitrile gradient in 0.1% formic acid using an analytical column PicoFrit Column (20 cm x ID75 μm, 5 μm particle size, New objective) at a flow rate of 300 nL/min over 180 min. The nanoelectrospray voltage was set to 2.1 kV and the source temperature was 275°C. All instrument methods for the LTQ Velos Orbitrap were set up in the data dependent acquisition mode of HCD fragmentation. The resolution in the Orbitrap system was set to r= 60,000 and the 5 most intense peptide ions with charge states ≥ 2 were sequentially isolated to a target value of 50,000 and fragmented in HCD with normalized collision energy of 40% with the resolution in the Orbitrap system was set to r= 7,500 for MS/MS. The signal threshold for triggering an MS/MS event was set to 80,000 counts and activation time of 0.1 ms was used. Dynamic exclusion was enabled with exclusion size list of 400 and exclusion duration of 60 s, and repeat count of 2.

Peak lists (msf) were generated from the raw data files using Proteome Discoverer version 1.3 (Thermo Fisher Scientific) with Sequest search engine and searched against NCBI database IPI Human v3.86 (IPI Human: 91,522 sequences; 36,630,302 residues, release July 2011) with carbamidomethylation (+57.021 Da), peptide N-terminus and lysine side chains with iTRAQ as fixed modification, oxidation of methionine (+15.995 Da) and tyrosine residues with iTRAQ as variable modifications, one trypsin missed cleavage and a tolerance of 10 ppm for precursor and 0.02 Da for fragment ions.

To ensure that only one PSM per spectrum is used for protein grouping it was applied strict maximum parsimony principle to protein grouping. Protein identifications were accepted if they contained at least one identified peptide. The peptides were filtered using cutoffs to obtain a false discovery rate less than 1%. For reporter ion identification, it was chosen the most confident centroid, which is almost always the largest peak inside the integration window because of the small inclination of the Gaussian curve in the 1-sigma interval. For reporter ion quantification, the validation step ensures that only peptides that have one of the specified reporter labels as a modification are considered for protein quantification and the protein ratios are calculate by the median of the peptide ratio. The mass spectrometry raw data are available *via* ProteomeXchange with identifier PXD019126.

### Bioinformatics and Statistics Analyses Workflow

In order to identify the differentially expressed proteins (DEP), we have established the following cut-off criteria: since we had to combine the samples into pools, this prevented a previous statistical analysis; therefore, the criteria used were the exclusion of the proteins that had no expression in the technical replicates and, arbitrarily, the fold-change values of 1.5 for the upregulated proteins and of 0.6 for the downregulated ones. In order to guarantee that there was no great variability among technical replicates, the coefficients of variation (CV) of the protein expression values were calculated and the CV cut-off criteria less than 15% was established to consider a DEP consistent. After identification of the DEP, we performed the bioinformatics analyses in order to extract relevant biological information from these proteins. The construction of the protein-protein interaction network and the subsequent analyzes were carried out separately for up and downregulated proteins, using the STRING tool, version 11.0 <https://string-db.org>. Then, the proteins that have no connection in the network were removed, and we performed the enrichment analyses to look for overrepresented biological function and pathways from GO (Gene Ontology) (20, 21) and KEGG (Kyoto Encyclopedia of Genes and Genomes) databases (22–25), respectively. P-value was adjusted for multiple comparisons by the FDR method (26) and values less than 0.05 was used as cut-off criteria to consider a category as significantly enriched. Finally, the function of the most relevant proteins was manually searched in the databases Gene <https://www.ncbi.nlm.nih.gov/gene>, from NCBI, and in the Gene Cards <https://www.genecards.org>, and we also made comparisons with data from previous transcriptomic and proteomic studies of our group, looking for possible overlapping of DEP. All graphs in this study, except for protein-protein interaction networks generated with the aid of STRING and Venn diagram generated with the help of <http://bioinformatics.psb.ugent.be/webtools/Venn/>, were constructed using the GraphPad Prism software version 8.0.

## Results

### Subjects

The clinical and laboratory characteristics of MM patients enrolled in this study (n = 8) are shown in **Table 1**. All patients were classified as advanced-stage MM according to the Durie & Salmon (27) and/or International Staging System criteria (28). Most were male (75%) and the median age at diagnosis was 68.5 years.

**Table 1:** Clinical and laboratorial characteristics of MM patients included in the study at diagnosis (N = 8).

Patients’ characteristics	
**Median age, years (range)**	68.5 (38-80)
**Sex, n (%)**	
Male	06 (75)
Female	02 (25)
**M-protein type, n (%)**	
IgG	04 (50)
IgA	03 (37.5)
Light chain	01 (12.5)
**Median tumor cells, % (range)**	75 (10-90)
**D&S^1^ stage n (%)** I	0 (0)
II	0 (0)
III	08 (100)
**ISS^2^ stage, n (%)**	
1	01 (12.5)
2	01(12.5)
3	06 (75)

^1^D&S, Durie & Salmon; ^2^ISS, International Staging System.

### MSC Isolation, Expansion, and Characterization

*In vitro* expansion of MSCs was successfully performed in four MM patients and four HD. MSC from both groups were positive for the markers assessed (CD105, CD90, and CD73), and negative for the markers (CD45, CD34, CD14, and HLA-DR), and there was no difference between groups **(** **Supplementary Figures 1** and **2** **)**. After *in vitro* induction for osteoblastic differentiation, it was also possible to detect osteocalcin in the medium of cell culture, and there were no difference among MM-MSC and HD-MSC in this parameter (data not shown, 16). Therefore, there were no differences between groups with regard to immunophenotype and osteoblastogenic potential after *in vitro* expansion.

### Protein Isolation, Quantification, and Mass Spectrometry Analyses

As described in the methodology, in order to obtain the protein concentration required for performing mass spectrometry analyses, the samples were pooled. Between MC^CD138-CD105-^, the protein concentrations from cases and controls were 67 and 99 μg, respectively, whereas between the MSC, the concentrations were 90 and 69 μg for cases and controls, respectively. 30 μg of protein from each group was used for iTRAQ kit labeling.

### Bioinformatic Analyses

Our study found 75 differentially expressed proteins (DEP) between MM-MC^CD138-CD105-^ and HD-MC^CD138-CD105-^, being 66 upregulated and nine downregulated. Regarding MM-MSC and HD-MSC comparison, our work found 14 DEP, being 12 upregulated and two downregulated. As mentioned in the methods, we chose CV values less than 15%, among technical replicates, to consider a DEP as consistent. With regard to both comparisons, 80% of the total DEP were consistent according to our cut-off. Detailed information on the up and downregulated proteins derived from both comparisons, can be seen in the supplementary material **(** **Supplementary Tables 1** and **2** **)** and in the **Table 2**.

**Table 2:** MM-MSC and HD-MSC comparisons detected 14 differentially expressed proteins, being 12 upregulated and two downregulated.

Status	Gene Symbol	Protein Description	115/114	114/115
Upregulated	*S100A11*	Protein S100-A11	14.442	1.930
	*HLA-A*	HLA class I histocompatibility antigen, A alpha chain	9.565	12.014
	*GAPDH*	Glyceraldehyde-3-phosphate dehydrogenase	5.138	1.823
	*VIM*	Vimentin	2.521	2.376
	*TALDO1*	Transaldolase	2.280	1.908
	*ANXA5*	Annexin A5	2.153	2.395
	*PKM*	Pyruvate kinase PKM	2.105	1.972
	*CANX*	Calnexin	1.766	1.642
	*PSMA1*	Proteasome subunit alpha type-1	1.736	1.599
	*ALPL*	Alkaline phosphatase, tissue-nonspecific isozyme	1.731	1.817
	*RAB7A*	Ras-related protein Rab-7a	1.645	1.782
	*CLTC*	Clathrin heavy chain 1	1.522	1.543
**Status**	**Gene Symbol**	**Protein Description**	**115/114**	**114/115**
Downregulated	*DPYSL2*	Dihydropyrimidinase-related protein 2	0.205	0.556
	*CTSZ*	Cathepsin Z	0.554	0.546

**Figure 1:**
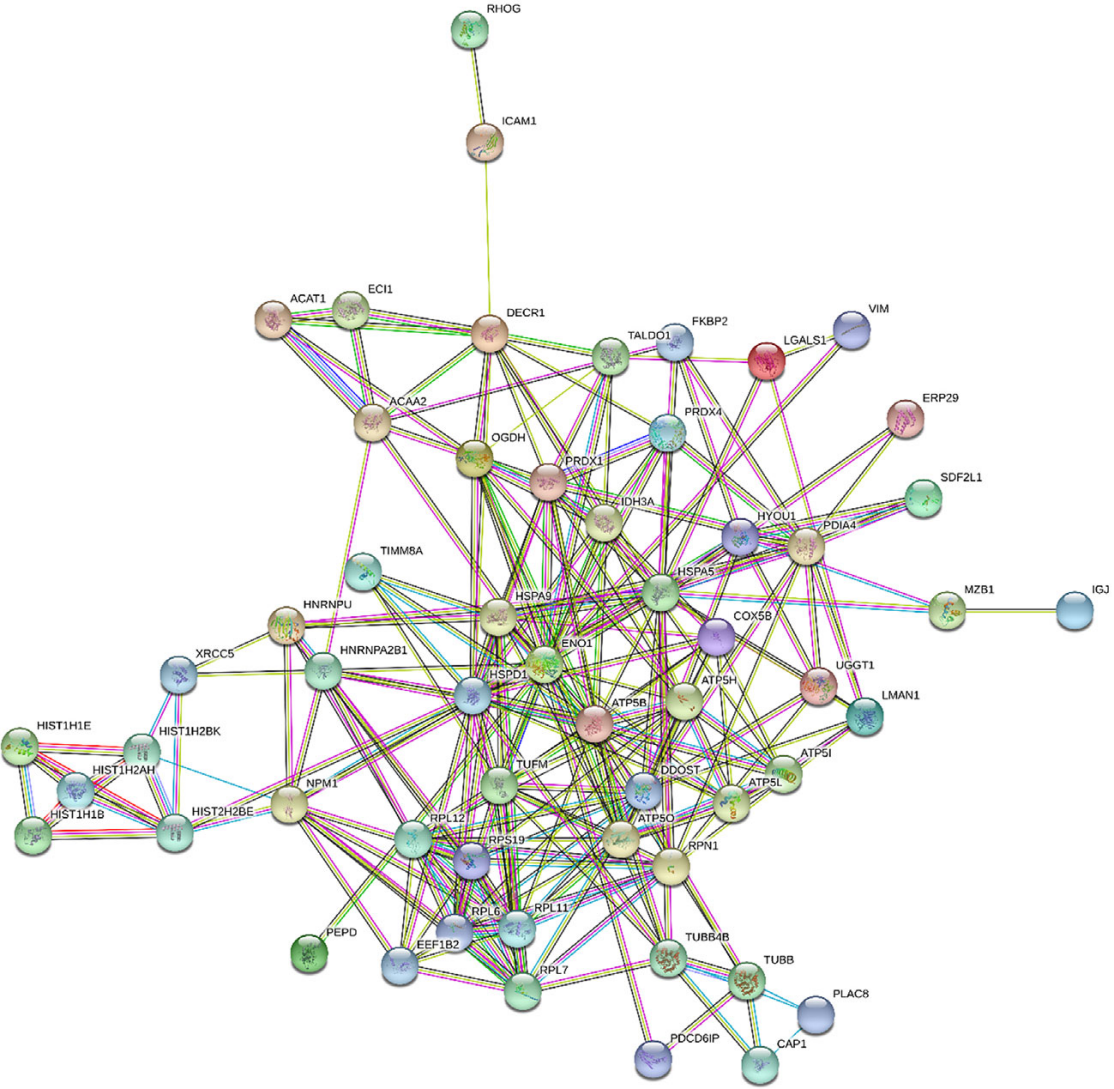
Protein-protein interaction network in MM- MC^CD138-CD105-^ *vs.* HD-MC^CD138-CD105-^: upregulated proteins. Nodes represent proteins and edges represent protein-protein associations. Node color: colored nodes and white nodes represent, respectively, query proteins and first shell of interactions, and second shell of interactions. Node content: empty and filled nodes represent, respectively proteins of unknown 3D structure, and some 3D structure is known or predicted. The edges are composed of known interactions, predicted interactions, among other forms of interaction, including co-expression. (STRING https://string-db.org/).

**Figure 2:**
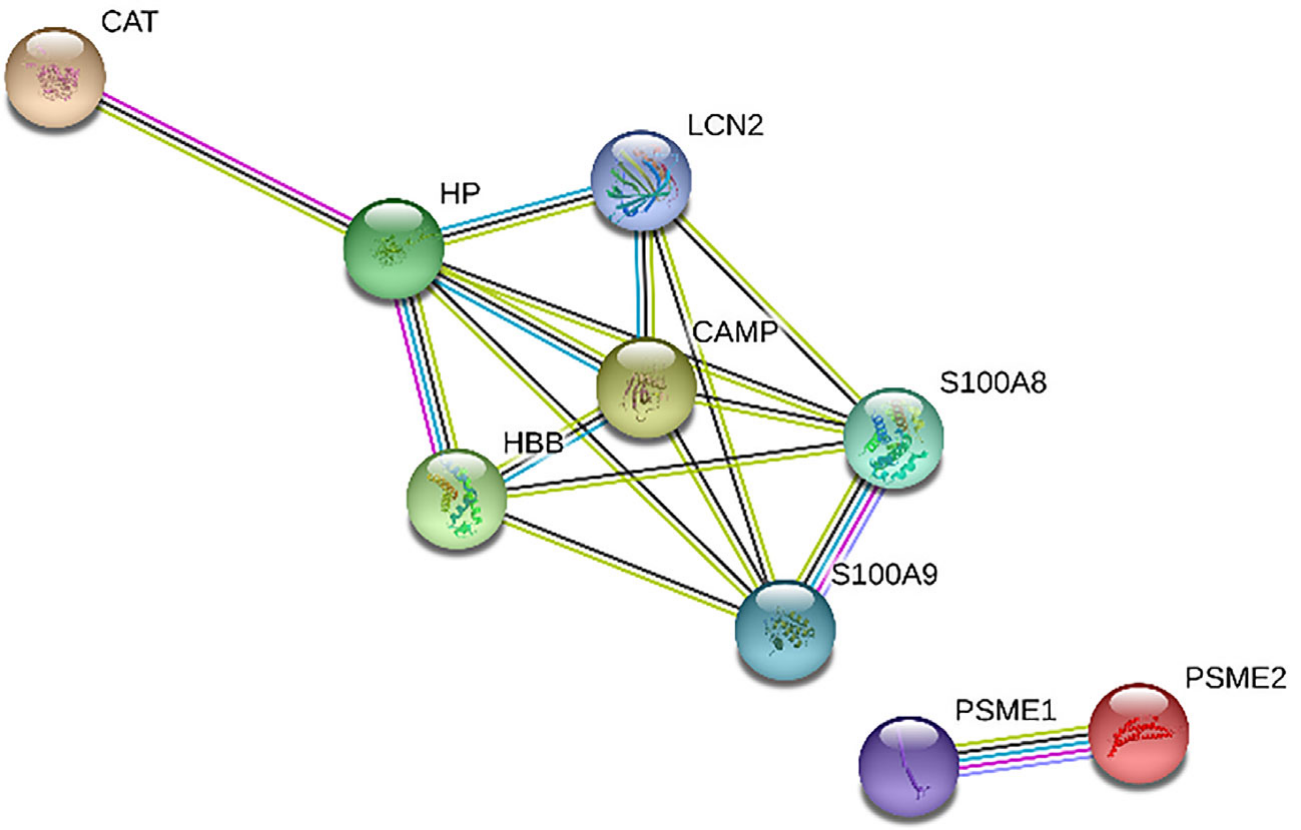
Protein-protein interaction network in MM-MC^CD138-CD105-^ *vs.* HD-MC^CD138-CD105-^: downregulated proteins. Nodes represent proteins and edges represent protein-protein associations. Node color: colored nodes and white nodes represent, respectively, query proteins and first shell of interactions, and second shell of interactions. Node content: empty and filled nodes represent, respectively proteins of unknown 3D structure, and some 3D structure is known or predicted. The edges are composed of known interactions, predicted interactions, among other forms of interaction, including co-expression. (STRING https://string-db.org/).

**Figures 1** and **2** show the protein-protein interaction networks for the up and downregulated proteins, respectively, between the MC of MM patients in comparison with the controls. Regarding MSC, due to the low number of DEP identified, we did not build the protein-to-protein interaction network for this comparison.

#### MM-MC^CD138-CD105-^ *vs.* HD-MC^CD138-CD105-^

**Figure 3:**
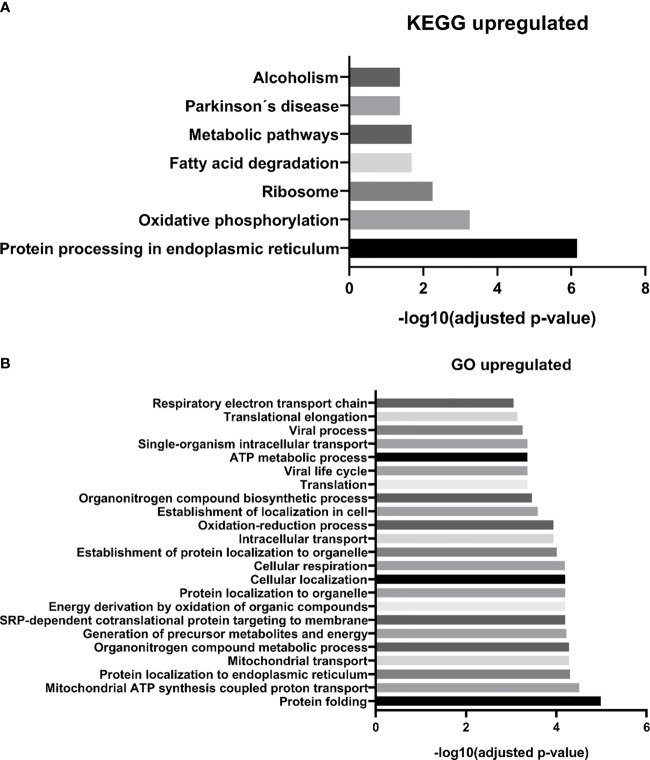
MM-MC^CD138-CD105-^ *vs.* HD-MC^CD138-CD105-^ upregulated proteins. – **(A)** KEGG enriched pathways **(B)** GO enriched biological processes.

We identified 68 biological processes of GO significantly unregulated between patients and controls (FDR <0.05). As the number of biological processes in the GO found was high, we chose to graphically represent only those with the highest level of significance (FDR <0.001), resulting in 24 processes **(** **Figure 3A** **).** Among the upregulated processes, we highlight three in particular - 1) Energy metabolism: which represented about 40% of the total proteins identified, including pathways related to cellular respiration and ATP synthesis (e.g.: “GO:0042776 mitochondrial ATP synthesis coupled proton transport”; “GO: 0046034 ATP metabolic process”; “ GO:0022904 respiratory electron transport chain”), oxidation reactions (e.g.: “GO:0015980 energy derivation by oxidation of organic compounds”; “GO:0055114 oxidation-reduction process”), and catabolic processes (e.g.: “GO:0009056 catabolic process”; “GO:0044248 cellular catabolic process”; “GO:1801575 organic substance catabolic process”), among others. 2) Cellular localization: representing about 13% of undetected processes, mainly pathways related to protein localization in specific regions of cells, such as “GO:0070972 protein localization to endoplasmic reticulum”. 3) Protein synthesis: approximately 20% of the total upregulated proteins found were related to protein synthesis, ranging from translation (e.g.: “GO:0006412 translation”, “GO:0006415 translational termination”), to protein folding, such as “GO:0006457 protein folding”, and “GO:0061077 chaperone-mediated protein folding”. We also identified seven biological pathways from the KEGG database significantly overrepresented **(** **Figure 3B** **),** in line with the GO biological processes, with emphasis on the following pathways: “protein processing in endoplasmic reticulum” and “ribosome” (both related to protein biosynthesis), and “oxidative phosphorylation”, “fatty acid degradation”, and “metabolism pathways “(all of them related to energy metabolism). In addition, we also identified an overlap of 24 upregulated DEP proteins from this study, compared to the data from our 2015 study (19), where we evaluated the protein expression of tumor plasma cells versus plasma cells from healthy donors **(** **Figure 5** **).**

**Figure 4:**
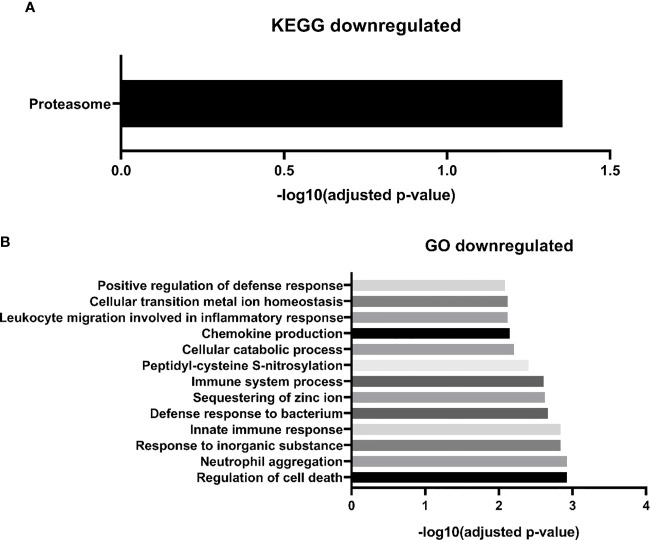
MM-MC^CD138-CD105-^ *vs.* HD-MC^CD138-CD105-^ downregulated proteins. – **(A)** KEGG enriched pathways **(B)** GO enriched biological processes.

Regarding downregulated proteins, we identified 27 significantly enriched biological processes. We chose to represent in **Figure 4A**, only the biological processes with the highest level of significance (FDR <0.01), resulting in 14 biological pathways. Most of the biological processes found by our study were related to the immune response, including the innate immune response, such as “GO:0070488 neutrophil aggregation”, “GO:0045087 innate immune response”, “GO:0030593 neutrophil chemotaxis”, among others. We also find fewer processes related to cell death, such as “GO:0010941 regulation of cell death”, “GO:0042981 regulation of apoptotic process”, and processes related to zinc ion homeostasis, including “GO: 0006882: cellular zinc ion homeostasis”. Finally, with regard to the biological pathways of the KEGG database, we found only one significantly enriched pathway “proteasome” **(** **Figure 4B** **).** Two downregulated proteins participate in this pathway: subunits 1 and 2 of the proteasome activator complex.

#### MM-MSC *vs.* HD-MSC

We found 14 DEP among the MSC of patients with MM compared to their normal counterpart from HD, being 12 upregulated and two downregulated **(** **Table 2** **).** Mainly due to the low number of DEP, no significantly enriched process or biological pathway was found in the GO and KEGG databases. Thus, we manually evaluated the 14 DEP through searches in the literature. In line with the results of the MC, among the upregulated proteins, some of them were involved in energy metabolism, such as glyceraldehyde-3-phosphate dehydrogenase, transaldolase 1, and pyruvate kinase M1/2, demonstrating a possible importance of the energy metabolism pathways in the pathophysiology of the disease. One upregulated protein that deserves to be highlighted is protein S100-A11. It is a member of S100 calcium-binding protein family, participating in the regulation of several cellular processes, including - but not limited to - cell cycle progression and differentiation. It has also been described in different types of cancer, playing an important role in tumorigenesis and metastasis. Another upregulated protein worth mentioning is calnexin. It is a protein that belongs to the calnexin family of molecular chaperones and plays an important role in the correct folding of proteins. Additionally, the upregulation of the proteasome subunit alpha type-1, which is a component of the proteasome complex and involved in the proteolytic degradation of intracellular proteins, has already been described in the literature in tumor plasma cells and may appear as a mechanism of resistance of these cells to effect of the proteasome inhibitor bortezomib. Finally, the RAB7A protein, member of the RAS oncogene family, was also upregulated in MM-MSC. It participates in several cellular processes, being able to perform its role as oncosuppressor or oncogenic.

## Discussion

The tumor microenvironment is extremely important for the development, maintenance and progression of different types of cancer, being MM one of the malignancies where its essential role has been demonstrated by several independent studies (12, 13). Despite its importance in the pathophysiology of MM, the BM tumor microenvironment of MM patients is still not so much studied. MSC are considered the most relevant player and, as a result, are the most studied cell type (29–33). However, other cell types present in BM, including lymphocytes, monocytes, dendritic cells, among others, also play an important role in the disease progression. Thus, this study aimed to analyze these cells more deeply, through a proteomic and bioinformatic analyses, in order to better understand their proteome expression profiling, aiming to identify DEP that may serve as potential biomarkers/therapeutic targets, and/or further elucidate the pathophysiology of MM. Besides, we also studied the proteome expression profiling of MM-MSC in comparison with HD-MSC. Protein expression profiling analysis of MM-MC^CD138-CD105-^ *vs.* HD-MC^CD138-CD105^ revealed 75 DEP, being 66 upregulated and nine downregulated. Bioinformatics analyses of these DEP showed that among upregulated proteins, the main functions and pathways were related to energy metabolism, cellular localization, and protein synthesis, whereas, among downregulated proteins, the main pathways and functions were related to the immune response, mainly to innate immunity, which raises the hypothesis of possible mechanisms of evasion of the immune response. Regarding MM-MSC vs. HD-MSC, we identified 14 DEP, being 12 upregulated and two downregulated. The search in the literature and databases showed that the most relevant proteins were among the upregulated proteins, with emphasis on the proteins S100-A11, calnexin, proteasome subunit alpha type-1, and RAB7A protein, all of them proteins described in the literature as being important in the pathophysiology of other types of cancer. Our study was the first to identify their increased expression in MM-MSC. In addition, other proteins upregulated in MM-MSC were also related to energy metabolism, as well as MM-MC^CD138-CD105-^, demonstrating a potential role of this pathway in the pathophysiology of the disease.

**Figure 5:**
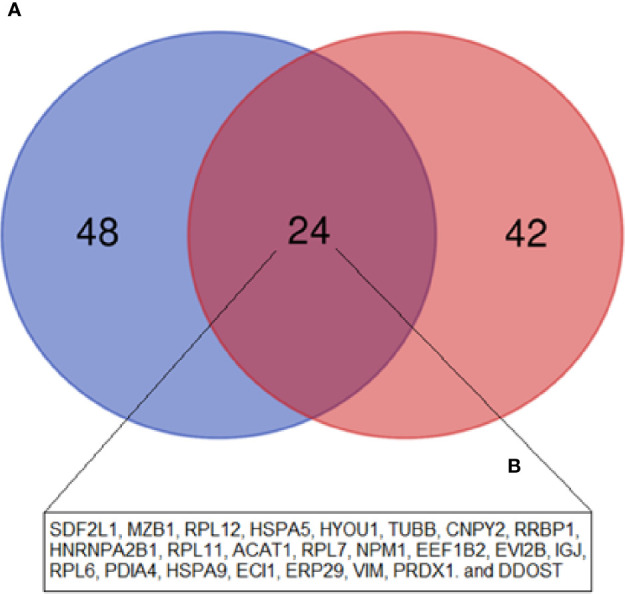
Venn diagram showing the overlap between the upregulated DEP in the current study (MM-MC^CD138-CD105-^ *vs.* HD-MC^CD138-CD105-^) compared to the upregulated DEP in our previous study (MM Plasma cells *vs.* HD Plasma cells) by Fernando et al. (19). **(A)** Fernando et al. (19) study; **(B)** Current study.

High throughput techniques, such as mass spectrometry, microarray, RNA-seq, among others, allow the generation of an abundant amount of data, allowing to compare patient cells in pathological conditions, such as MM, in relation to cells derived from HD. In 2015, our group published a proteomics study in which we evaluated the protein expression of tumor plasma cells from MM patients in comparison to plasma cells from palatal tonsils from normal individuals (19). In this study, we identified 81 DEP among these cells (72 upregulated and nine downregulated). Among upregulated proteins, we highlighted the ones related to protein biosynthesis, while among downregulated proteins, most of them were related to the immune response. These results are in line with what we identified in this present study between MM-MC^CD138-CD105-^ and HD-MC. Regarding the upregulated proteins from these two studies, we do see an overlap of 24 proteins (**Figure 5**). Among them, there are mainly proteins related to protein biosynthesis, including chaperones, for instance, the endoplasmic reticulum chaperone BiP, and hypoxia upregulated 1, ribosomal proteins, and proteins capable of post-translational modifications. On the other hand, among the downregulated proteins, no overlap was identified. Our group also published another paper in 2019, where we evaluated the MM-MSC transcriptome compared to HD-MSC (16). In this study, we identified 485 differentially expressed genes (280 up and 205 downregulated). The most significant pathways and functions were among the downregulated genes, especially genes involved in cell cycle progression, immune response, and bone metabolism. However, in this case, we did not identify any overlap with the proteins identified in this study.

In relation to upregulated DEP identified in the MM- MC^CD138-CD105-^ *vs.* HD-MC^CD138-CD105-^ comparison, energy metabolism was one of the enriched pathways identified through bioinformatics analysis. Ward and Thompson (34) have proposed that metabolic reprogramming, driven by activated oncogenes and inactivated tumor suppressors, is a cancer hallmark. Metabolic changes have already been described in different types of malignancies, including MM - increase in glycolysis and glutaminolysis in MM cells compared to healthy cells, among other changes (35). Some studies have demonstrated the role of these metabolic alterations in drug resistance in patients who are refractory to current available treatments (36). In another study, the authors evaluated the proteome of plasma cell from naïve MM patients, in order to identify pathways associated with good response to bortezomib-based treatment protocols. The authors reported that alternation in energy metabolism might be associated with good response to a specific bortezomib-based regime (37). Recently, Ray et al. (38) have identified Alpha-Enolase (*ENO1*), which was one of the DEP detected in our study in the mononuclear cells comparison, as a possible immunometabolic target in MM. The authors have demonstrated, through *in vitro* analyses, that BM plasmacytoid dendritic cells (pDCs) from MM patients induce *ENO1* expression in MM cells and pDCs, and they also have showed that patients with high *ENO1* expression have poor overall survival in comparison with the ones with low expression of this gene. In addition, they also reported that the inhibition of *ENO1* induces the activation of CD8^+^ T cells and NK cells against MM cells. Another protein, IDH3A, which was connected with ENO1 in our protein-protein interaction network, might also be important for MM pathogenesis. Although its role in malignant tumors is still unclear, its overexpression was reported in hepatocellular carcinoma and in extramedullary plasmacytomas (39, 40). In addition, other two proteins, which were also connected with ENO1 in our network, might play important roles in MM: Oxoglutarate dehydrogenase (OGDH) – it is a subunit of an enzymatic complex that participates in the Krebs cycles, and it was also found that several cancer cells depend on this protein for growth and survival (41); Heterogeneous nuclear ribonucleoprotein A2/B1 (hnRNPA2B1) – it influences pre-RNA process, and mRNA metabolism and transport; Shen et al. (42) have demonstrated that a long non-coding RNA, ST3GAL6-AS1, might promote adhesion and invasion of MM cells, through binding with hnRNPA2B1, resulting in the regulation of ST3GAL6 expression (42). Therefore, studies aiming to better understand the metabolic changes, both in the tumor plasma cells as well as in the tumor microenvironment, are extremely important in the search for new therapeutic targets, especially for refractory patients.

Regarding protein biosynthesis, which was another category identified among upregulated DEP in MC, we highlight proteins related to “protein processing in endoplasmic reticulum”, namely the following genes that code for these proteins *RRBP1*, *ERP29*, *PDIA4*, *LMAN1, DDOST, UGGT1, RPN1, HSPA5*, and *HYOU1*, and the ones related to “protein folding”, which are *PRDX4, FKBP2, ERP29, PDIA4, LMAN1, HSPA5, UGGT1, HSPD1, HSPA9*, and *GNB2*. The HSPA5, HSPA9, and HYOU1 proteins are part of the heat shock protein 70 family, a family of chaperones involved in physiological processes, such as the assistance of general folding of unfolded and/or misfolded proteins, and which has been described as deregulated in MM patients (43, 44). Another chaperone was also identified - HSPD1 - which belongs to the HSP60 family. Recently, the HSPD1 protein has been described to regulate metabolic and protein synthesis changes in MM cells, promoting its proliferation, thus representing a potential therapeutic target (45). Moreover, we found ribosomal proteins, including RPL6, RPL7, RPL11 and RPL12, which form the 60S subunit of ribosomes, and RPS19, which form the 40S subunit. In summary, we can highlight the relevance of chaperones belonging to the HSP70 and HSP60 families, some of which have been already described by other studies as deregulated in the tumor plasma cells of patients with MM, contributing to disease progression by maintaining protein homeostasis, blocking apoptosis and helping in the stabilization of oncoproteins (44). In addition, early clinical and preclinical studies have shown that HSP inhibitors, especially inhibitors of the HSP90 family, can be promising therapeutic targets in the treatment of patients with MM (46).

In healthy individuals, plasma cells are terminally differentiated B cells which are responsible for the synthesis of immunoglobulins against pathogens. Due to the high production of proteins, these cells are naturally subject to endoplasmic reticulum (ER) stress, which activates a pathway known as UPR, helping the cell to overcome this type of stress (47). In the case of MM patients, the tumor plasma cells, in the vast majority of patients, produce a monoclonal immunoglobulin, known as paraprotein. To deal with this type of stress, that is, an extremely high production of immunoglobulin, the UPR pathway is very and constantly activated and allows the tumor cell to remain viable (47). In the case of cells in the tumor microenvironment, paraprotein is not produced. On the other hand, due to the action of tumor plasma cells on these cells, there is a production of different types of soluble elements, such as cytokines, chemokines and growth factors (13). One hypothesis is that proteins related to protein biosynthesis, as well as UPR, as is the case of chaperones, are important to keep these cells viable by producing molecules important for the maintenance and progression of MM cells. Therefore, our hypothesis is that other MM BM mononuclear cells contribute to maintenance of tumor plasma cells homeostasis, avoiding tumor cell death due to accumulation of cytoplasmic immunoglobulin overload. Other possibility is that this process is too important for MM tumor cells that, even in a more than 85% enriched pool of MCs, we can still see these proteins’ expression as a sign of residual tumor plasma cells contamination.

Regarding downregulated proteins in the MC “compartment”, proteasome activators complex subunits 1 and 2 proteins are components of the proteasome and play an important role in its regulation. In bortezomib-resistant patients (a class of proteasome inhibitor used in the treatment of MM), these proteins are upregulated (37). Therefore, these protein downregulation in MCs is totally in agreement with their function, not related to overexpression of proteasome pathway typically found in MM tumor cells.

Still in relation to MM-MC^CD138-CD105-^, most of the downregulated proteins were related to the immune response, such as antigen processing, leukocyte recruitment, production of pro-inflammatory cytokines, and immune responses against pathogens. We can highlight the downregulation of proteins S100-A9 and S100A9, which form a complex called calprotectin and are involved in different functions, for instance, enhancing leukocyte recruitment and pro-inflammatory cytokines release. HP and CAMP have antimicrobial properties, whereas PSME1 and PSME2, discussed above, are also required for efficient antigen processing. The decrease in these proteins involved in the immune system may be related to mechanisms of tumor immune evasion, which have also been described by other groups (48, 49). We also highlight the downregulated of pathways related to innate immune response. Although the innate immune response is not as studied as adaptive response in MM patients, some studies have already demonstrated important changes in this arm of the immune response, such as changes in genes which code for innate immune response proteins, increasing the risk of developing MM (50). Additionally, Magalhães et al. (51) demonstrated that patients with long-term disease control and those with symptomatic MM differ, among other parameters, in the number of dendritic cells and tissue macrophages in BM. In addition, the number and function of NK cells is impaired in MM, and the function of dendritic cells is also altered (52). Among the new strategies to treat refractory MM cases are CART-cells and BiTEs (11). Chimeric antigen receptors (CARs) are chimeric proteins that assemble the signaling portions of the T cell receptor complex (TCR) and the variable domains of an antibody targeting an antigen of interest. A variety of targets are currently being studied in MM and include BCMA, SLAMF7, CD138, NKG2DA, kappa light chain, and CD19 ligands. Ongoing clinical trials will define its role in the coming years (11, 53). BITEs are generated to combine the specificities of two antibodies, binding simultaneously to multiple epitopes, one of which involves the activation of T cells through their CD3 molecules (54, 55). These new immunotherapies corroborate the relevance of microenvironment and strength immune system to control of refractory hematological tumors.

Finally, in relation to MM-MSC, although we have identified a low number of DEP, among the upregulated proteins, some of them are quite interesting and had not yet been described in the context of MM: Ras-related protein Rab-7a, among other functions, participate in cellular processes such as autophagy, apoptosis, signaling, and cell migration, being important for the progression and resistance to drugs in some types of cancer (56). Thus, our study suggests that further studies are needed to evaluate this molecule as a potential therapeutic target in the context of MM. In addition, we have also identified the protein S100-A11, which is a protein often upregulated in different types of human cancer, and its potential as a therapeutic target has not been evaluated yet in patients with MM. Besides, our analyses also found increased expression of the protein calnexin, a protein induced by stress in the endoplasmic reticulum. This protein has already been described in the literature as a prognostic marker and potential therapeutic target in colorectal cancer, but it has not yet been evaluated in MM (57). Finally, another protein that deserves attention is annexin V, which was upregulated in MM-MSC. Overexpression of annexin II, a protein that is related to annexin V, has already been described in plasma cells of MM patients and, in some cell lines of this disease, seems to promote proliferation, apoptosis, invasion potential and production of proangiogenic factors (58). In line with this data, Glavey et al. (59) reported that the upregulation of annexin II protein, produced by BM extracellular matrix from MM patients, is associated with a decreased overall survival.

The main limitations of this study are the low number of patients with MM enrolled. MM is a rare disease, representing only 1% of cases of cancer (3); in addition, all cases included here were seen at our public tertiary university hospital, the majority of them being severely ill patients, with advanced disease stage, often requiring immediate treatment due to medical emergencies. Since steroids or bisphosphonates could potentially alter protein expression of tumor microenvironment cells, patients who received these drugs were not eligible for the study. A possible source of bias is related to the BM biopsy site: in our study, we performed bone marrow biopsy in only one region of the iliac crest of each patient or control. However, recently, a group has demonstrated substantial intra-patient spatial heterogeneity in the bone marrow of MM patients (60). Another limitation that deserves to be highlighted is the fact that the case and control groups were not matched by sex or age, which might introduce bias in our analysis, including the relevant downregulation of immune system proteins related to physiological immunosenescence. However, several proteins identified as differentially expressed between the cells of these groups, had already been described by other groups as deregulated in MM, which increases the robustness of our data (61–63). In addition, another limitation of our study is the fact that the protein samples were analyzed in pools. This approach was necessary to obtain enough protein for the analysis proposed in this study. Although we performed experiments in technical replicates, which allowed us to verify the technical variability, we were unable to analyze possible biological variations among patients with MM. Finally, we also did not perform any functional analyses of the pathways/proteins deregulated by MM identified in our study. However, the main objective of our work was to identify molecules which can be better studied in the future as biomarkers and therapeutic targets: shifts in metabolic pathways and decrease of immune functions are among the cancer hallmarks ready to be explored in MM, which is representative of a cancer of immune system affecting elderly patients.

In summary, our study demonstrated that the BM tumor microenvironment of MM patients have a different protein expression profiling when compared to the BM microenvironment of healthy individuals. Among the main altered biological pathways in MC, we can highlight the increased expression of proteins related to different functions of protein biosynthesis, especially chaperone molecules, which have been studied as potential therapeutic targets in the treatment of MM in the last years. In addition, the increased expression of proteins related to energy metabolism, which was also identified by our work, has been increasingly studied in the context of different types of cancers, including MM, being a promising therapeutic target as well. The downregulation of different proteins involved in the immune response, may contribute to escape to the maintenance and progression of the disease, in addition to drug resistance. Thus, the new immunotherapies that have been studied for the treatment of MM and other cancers, such as CART-cells and BiTEs, can be promising tools to reverse this scenario of BM immunosuppression which MM patients are subjected. Finally, with respect to MM-MSC, we have identified some potential new therapeutic targets that can be evaluated in future studies.

## Data Availability Statement

The datasets presented in this study can be found in online repositories. The names of the repository/repositories and accession number(s) can be found in the article/**Supplementary Material**.

## Ethics Statement

The studies involving human participants were reviewed and approved by Institutional Review Board from Federal University of São Paulo. Written informed consent to participate in this study was provided by the participants’ legal guardian/next of kin.

## Author Contributions

RF: Data analysis and interpretation, and manuscript writing. FC: Study design, data collection, and review and approval of the final version of the article. AL: Data collection and analysis, and approval of the final version of the article. GC: Study design, data analysis and interpretation, and manuscript writing. All authors contributed to the article and approved the submitted version.

## Funding

This work was supported by Fundação de Amparo à Pesquisa do Estado de São Paulo, FAPESP (grant number 2010-17668-6/2017-21801-2).

## Conflict of Interest

The authors declare that the research was conducted in the absence of any commercial or financial relationships that could be construed as a potential conflict of interest.
