# Supplementary material for: Tumor Microenvironment Proteomics: Lessons From Multiple Myeloma
Source: Front Oncol. 2021 Mar 23;11:563384. doi: 10.3389/fonc.2021.563384 (PMC8021918; doi:10.3389/fonc.2021.563384)
Supplement: Supplementary file 2 [file DataSheet_2.docx]

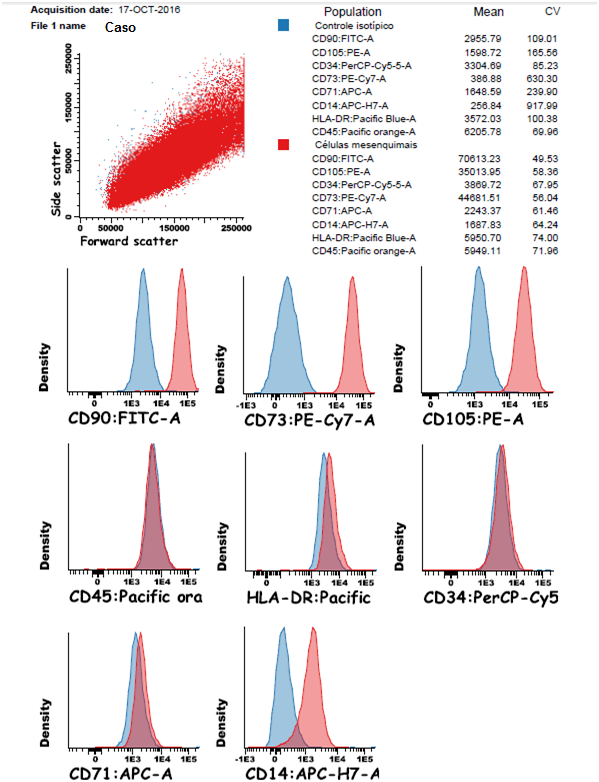


**Supplemental Figure 1. Forward Scatter (FSC) and Side Scatter (SSC) scatterplot, as well as the histograms containing the fluorescence intensities of the MM-MSC antigens of a representative patient (case) with MM (red color) and their respective isotypic controls (blue color).**


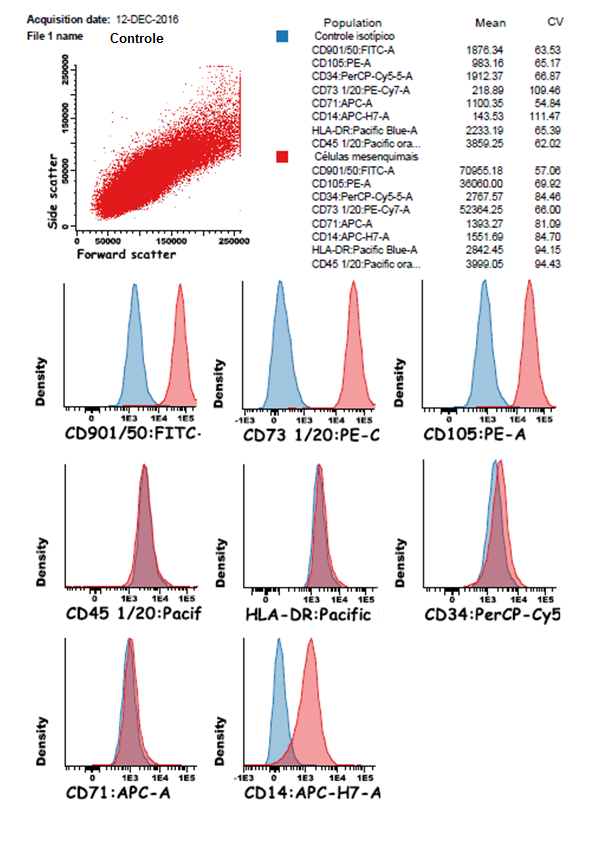


**Supplemental Figure 2. Forward Scatter (FSC) and Side Scatter (SSC) scatterplot, as well as the histograms containing the fluorescence intensities of the antigens evaluated in HD-MSC of a representative healthy individual (control, red color) and their respective isotypic controls (blue color).**
